# Ultrasound‐guided injections for the retrotrochanteric region: A cadaveric investigation

**DOI:** 10.1002/pmrj.13381

**Published:** 2025-05-12

**Authors:** Kamal Mezian, Vincenzo Ricci, Ke‐Vin Chang, Nimish Mittal, Jan Vacek, Ondřej Naňka, Levent Özçakar

**Affiliations:** ^1^ Department of Rehabilitation Medicine, First Faculty of Medicine Charles University and General University Hospital Prague Czech Republic; ^2^ Physical and Rehabilitation Medicine Unit, Luigi Sacco University Hospital A.S.S.T Fatebenefratelli‐Sacco Milan Italy; ^3^ Department of Physical Medicine and Rehabilitation National Taiwan University Hospital, Bei‐Hu Branch Taipei Taiwan; ^4^ Department of Physical Medicine and Rehabilitation National Taiwan University College of Medicine Taipei Taiwan; ^5^ Temerty Faculty of Medicine, Division of Physical Medicine and Rehabilitation University of Toronto Toronto Canada; ^6^ Department of Physiotherapy and Rehabilitation Faculty of Medicine Masaryk University Brno Czech Republic; ^7^ Institute of Anatomy, First Faculty Of Medicine Charles University Prague Czech Republic; ^8^ Department of Physical and Rehabilitation Medicine Hacettepe University Medical School Ankara Turkey

## Abstract

**Background:**

Injections for the piriformis and triceps coxae tendons/bursae have not been described and validated.

**Objective:**

To investigate the deep retrotrochanteric anatomy and validate an ultrasound (US)‐guided injection technique.

**Methods:**

Fifteen sides of the pelvic half/lower limb of formalin‐fixed cadaveric specimens were dissected to investigate the deep retrotrochanteric region. An US‐guided superficial/peritendinous green latex dye injection was performed on both sides of a single full body cadaver. Next, seven sides of another four full body cadavers were injected using a deep/intrabursal technique. The cadavers were dissected to observe the dye's location.

**Results:**

In the anatomical part of the study, we observed a consistent fusion of the piriformis tendon (PT) with the triceps coxae tendon (TCT) (15/15), and with the gluteus medius tendon in 93% (14/15). A bursa of piriformis was identified in 80% (12/15) of the cases, and a subtendinous bursa of obturator internus was found in 73% (11/15). A fat pad overlying the PT–TCT was present in 93% (14/15) of the cases. Regarding the US‐guided injections, success rate for superficial/peritendinous injection was 0% (0/2), with the latex dye being identified in the fat pad covering the PT–TCT in both cases. For the deep/intrabursal injection, the success rate was 86% (6/7).

**Conclusions:**

The results indicated a satisfactory success rate for the deep/intrabursal injection of PT–TCT in retrotrochanteric pain syndrome. This technique holds promise for the treatment of bursa and tendon pathologies in relevant patients.

## INTRODUCTION

Pain in the gluteal region is a common and debilitating condition that affects athletes and the general population. Potential causes of buttock pain are diverse, ranging from conditions within the gluteal region itself to referred pain from the lumbosacral spine, pelvis, hip, or thigh.^1^ Some patients experience more pronounced pain in the retrotrochanteric region, specifically at the insertion of the piriformis tendon (PT) and the triceps coxae tendon (TCT), which consists of the tendons of the superior gemellus (SG), obturator internus, and inferior gemellus.

These tendons are heavily involved in various physical activities, particularly in a weight‐bearing limb. Repetitive overload can lead to overuse tendinopathy^2^ and inflammation of the associated bursae.^3^ These tendons/bursae have been proposed as pain generators, but typically appear normal on magnetic resonance imaging/ultrasound (US) imaging. Targeted US‐guided injections of these structures could help confirm or rule out their involvement in painful conditions of the retrotrochanteric region. Smith and colleagues previously described and validated an US‐guided injection technique focused on the obturator internus, specifically targeting the tendon/muscle at a more medial location, without addressing the PT.^4^ Considering that optimal treatment outcome for TCT and PT tendinopathies requires a targeted approach, we describe an US‐guided injection technique for the deep retrotrochanteric space. To facilitate understanding of this technique, we also provide a detailed anatomical description of the region based on cadaveric dissection.

## MATERIALS AND METHODS

### Cadaveric Specimen

This study utilized five full body human cadavers (all female) embalmed with the Fix for Life (F4L) method (Table 1).^5^ This method was already shown to be suitable for US‐guided injections.^6^, ^7^ For anatomical evaluations, an additional 15 specimens, originally used in medical student dissection classes, were prepared using the standard formaldehyde embalming method (Table 2). Informed consent had been obtained from donors for scientific and educational purposes prior to death, and approval was obtained from the Institute of Anatomy, First Faculty of Medicine, Charles University, Prague. Ethics committee or institutional review board approval was not required for this study. All dissections were performed by an academic anatomist (O.N.) with over 25 years of experience.

**TABLE 1:** Cadaveric features and postinjection dissection findings (latex dye localization) for the deep/intrabursal approach.

Cadaver	BMI	Gender	Right side	†	Left side	†
Deep/intrabursal approach						
Specimen 1	27	F	Intrabursal	Sciatic nerve	Intrabursal	Deep to TC
Specimen 2	32	F	N/A		Intrabursal	Superficial to TC
Specimen 3	18	F	Intrabursal	Trochanteric bursa	Intraarticular	
Specimen 4	20	F	Intrabursal	Superficial to TC	Intrabursal	Deep to GM
Superficial/peritendinous approach						
Specimen 5	30	F	Intraadipose		Intraadipose	

Abbreviations: **†**, sites where the dye was also found; BMI, body mass index; F, female; GM, gluteus medius; M, male; N/A, not available; TC, triceps coxae.

**TABLE 2:** Dissection features of the piriformis muscle/tendon and the triceps coxae.

Cadaveric specimen	Gender	Side	Fusion of the PT with gluteus medius	Fusion of the PT with triceps coxae	Overlying fat pad presence	Subtendinous bursa of obturator internus	Bursa of piriformis	Piriformis muscle variations	Sciatic nerve division
1	M	L	+	+	+	+	+	Normal	Normal
2	F	R	+	+	+	−	−	Normal	Normal
3	M	L	+	+	+	+	+	Bifurcation	High
4	F	R	+	+	+	+	+	Normal	Normal
5	M	L	+	+	+	−	+	Normal	Normal
6	F	L	+	+	+	+	−	Normal	Normal
7	F	R	+	+	+	+	+	Normal	Normal
8	F	R	+	+	+	+	+	Normal	Normal
9	F	R	+	+	+	+	+	Normal	Normal
10	F	R	+	+	+	+	+	Normal	Normal
11	F	L	−	+	−	−	−	Normal	Normal
12	M	L	+	+	+	+	+	Bifurcation	High
13	F	L	+	+	+	−	+	Normal	Normal
14	F	R	+	+	+	+	+	Bifurcation	High
15	M	R	+	+	+	+	+	Bifurcation	High

Abbreviations: F, female; L, left; M, male; PT, piriformis tendon; R, right.

### Dissection – Anatomy

In the posterior buttock region, the superficial layers of skin, subcutaneous tissue, and the gluteus maximus muscle had already been removed or dissected during previous medical education sessions. In most cases, we needed to extend the preexisting dissections of the gluteus maximus. First, the sciatic nerve was identified and traced proximally until the piriformis muscle was clearly visible. The piriformis muscle was then traced toward its tendinous insertion at the greater trochanter. To elucidate the deep retrotrochanteric region, the fat pad covering the piriformis and the triceps coxae was removed. Anatomical tweezers were used to elucidate the different muscles and their insertional anatomy. We noted anatomical variations of the sciatic nerve and the piriformis muscle belly. The relationship between the PT, the triceps coxae (SG, obturator internus, and inferior gemellus), and gluteus medius muscles were also variable. Subsequently, the piriformis and triceps coxae tendons were dissected. Beneath these tendons, the space superficial to the posterior hip joint capsule was explored to assess the presence of the bursa of piriformis and the subtendinous bursa of obturator internus.^8^, ^9^

### Ultrasound‐guided Injection

**FIGURE 1:**
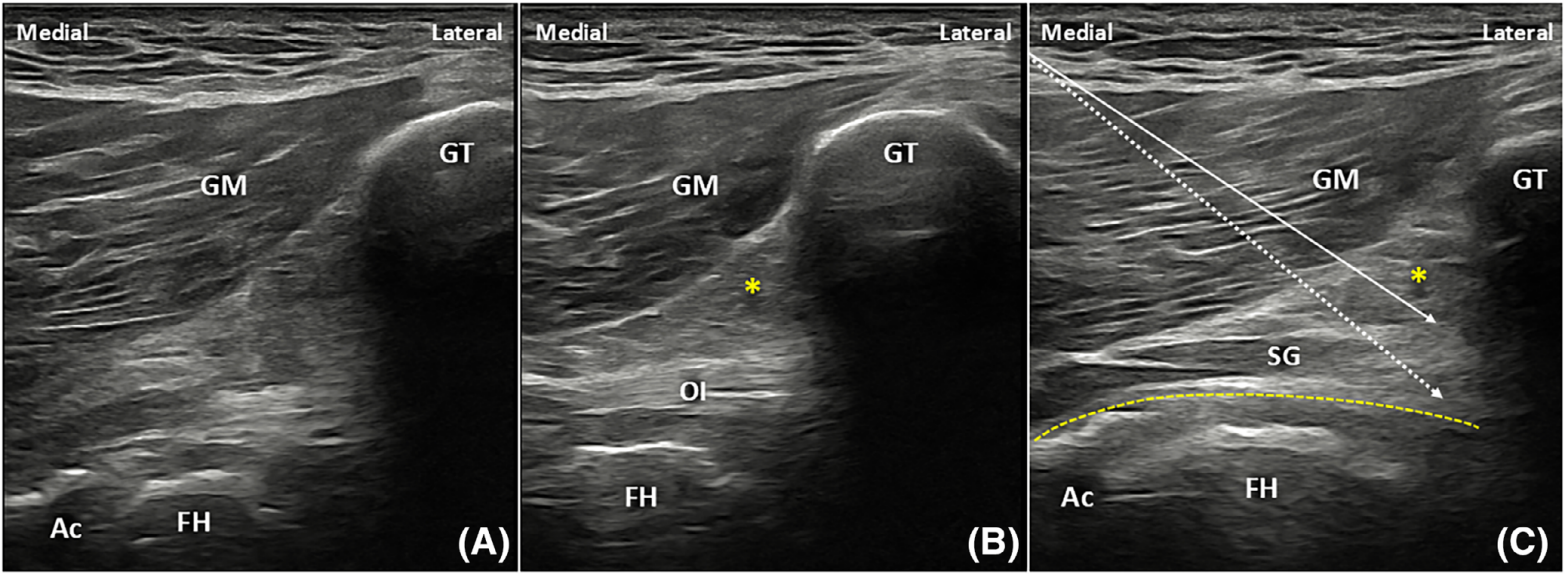
Sonographic planning of the procedure demonstrated on a healthy male individual, aged 41 years. A transverse scan over the greater trochanter serves as the initial image. Moving the probe slightly medially, the retrotrochanteric area becomes visible (A), with the femoral head (FH) and acetabular labrum (Ac) depicted on the screen. As the transducer is moved slightly cranially (B), the obturator internus tendon (OI) appears as a well‐defined fibrillar structure. Finally, for the injection, the probe is moved slightly more cranially (C), where the superior gemellus (SG) becomes the first muscular structure to appear after the OI disappears. The *dashed arrow* represents the needle's pathway deep in the interface between the posterior hip joint capsule and the triceps coxae and piriformis tendons (situated more cranially, out of view), and the *solid arrow* indicates the needle path superficial to the piriformis and triceps coxae. Asterisk indicates a fat pad; the curved dotted line indicates the hip joint capsule. GM, gluteus maximus; GT, greater trochanter.

A single physiatrist (K.M.) with over 10 years of experience in US‐guided musculoskeletal procedures performed all the injections on the five full body cadavers positioned prone on the dissection table. All procedures were conducted using a 38‐mm footprint, 3–16 MHz linear phased array transducer (UGEO HM70A, Samsung, Seoul, South Korea). A transducer cover sheet was used with standard colorless US gel. The transducer was positioned in the transverse plane over the lateral aspect of the hip to identify the greater trochanter.^10^, ^11^, ^12^ The US probe was then moved slightly medially to visualize the posterior facet of the greater trochanter (Figure 1A). Subsequently, the transducer was moved cranially until a well‐defined tendinous structure representing the obturator internus appeared on the screen (Figure 1B). The probe was then slightly moved further cranially until the obturator internus disappeared from the screen and the first muscular structure, representing the SG, was identified. The most cranial level of the SG was considered optimal for injection, in order to minimize the risk of passage through the obturator internus tendon. Before the injection, the course of the sciatic nerve was observed to avoid possible injury. An in‐plane medial‐to‐lateral approach was used, with 2 mL of 50% tap water‐diluted green latex dye injected at the target site in all cases. A heel‐toe maneuver with the needle was employed to enhance its visibility.

This study utilized two injection approaches: superficial/peritendinous and deep/intrabursal. Initially, the latex dye was injected superficial to the PT–SG complex (Figure 1C) to achieve peritendinous dye distribution. For the intrabursal technique, the needle tip was placed deeply, between the PT–SG complex and the hip joint capsule (Figure 1C).

### Dissection – Postinjection

First, the skin was removed to expose the gluteus maximus, which was subsequently dissected using a scalpel. In selected cases, to enhance visualization of the tendon attachment area, we performed an incision along the fiber direction of the gluteus maximus and retracted the muscle using hooks. The sciatic nerve and piriformis muscle belly were then identified. The piriformis was traced toward its insertion at the greater trochanter by removing the covering fat pad. Tweezers were used to separate the individual parts of the triceps coxae, piriformis, and gluteus medius tendons. Dye placement was considered intrabursal if visualized in the interface between the posterior hip joint capsule and the TCT and PT and peritendinous if the dye was present superficial to the aforementioned tendons.

### Statistical analysis

The statistical analysis conducted in this study is primarily descriptive, with the main outcomes in percentages and exact numerical values for individual observations.

## RESULTS

### Anatomic Variations

**FIGURE 2:**
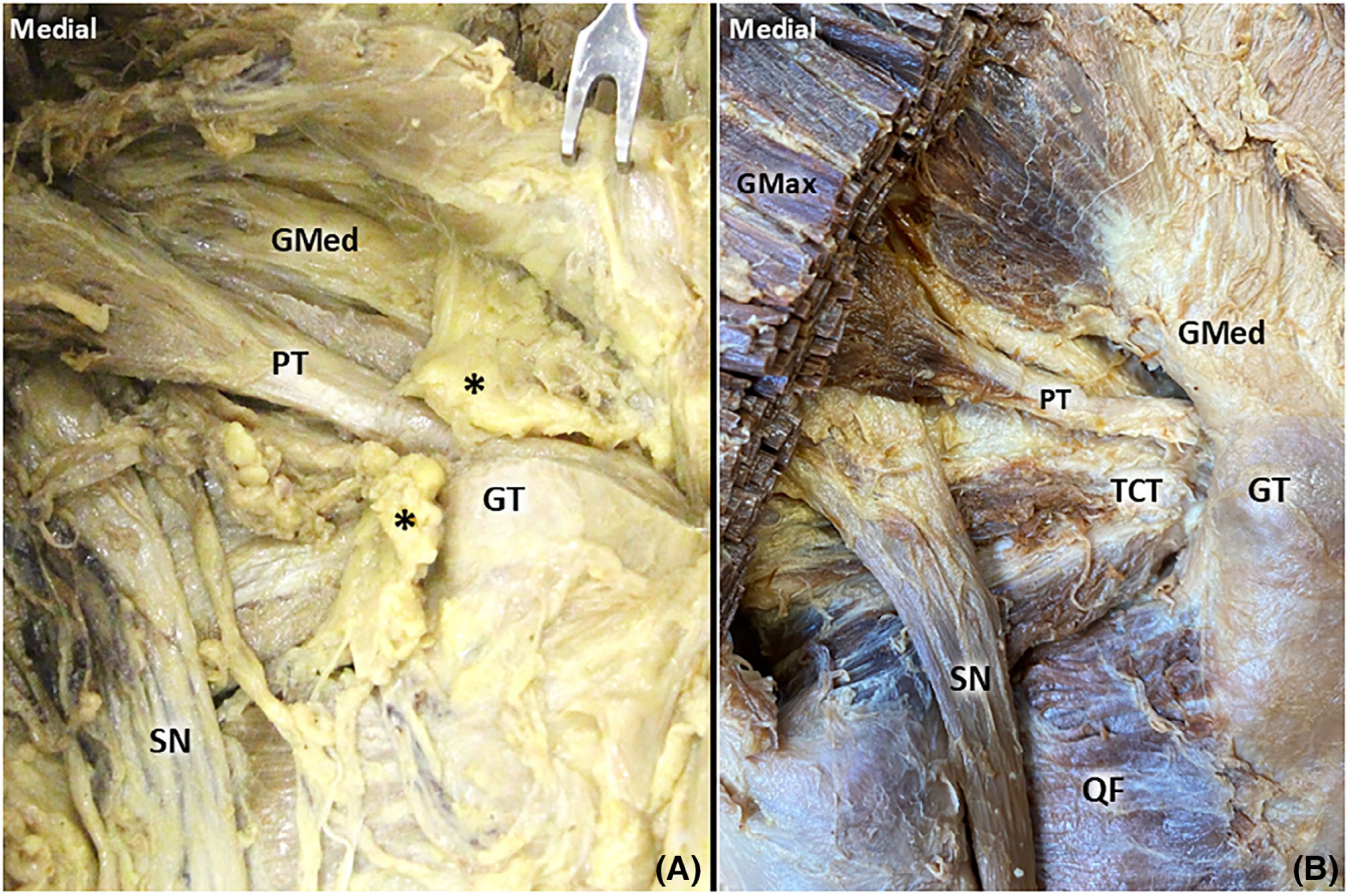
Anatomy of the retrotrochanteric region – superficial layer. After removal of the skin and dissection the gluteus maximus muscle (GMax), the surrounding fat pad *(black asterisk)* overlying the piriformis tendon (PT) and triceps coxae (TCT) becomes visible (A). When the fat pad is removed, the attachment of the PT and TCT at the greater trochanter (GT) is clearly seen (B). GMed, gluteus medius; QF, quadratus femoris; SN, sciatic nerve.

**FIGURE 3:**
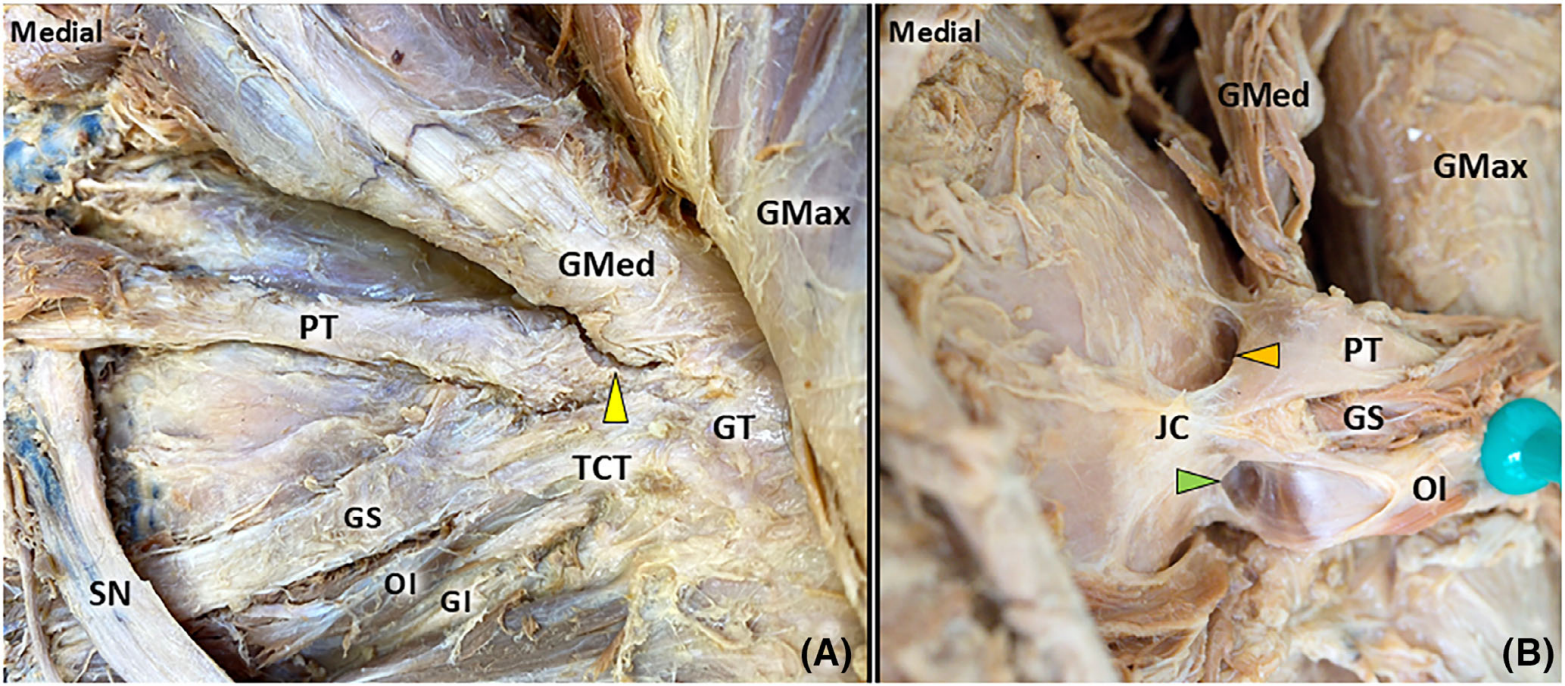
Anatomy of the retrotrochanteric region – deep layer. During the sharp separation of the piriformis tendon (PT) from the gluteus medius (GMed) using a scalpel, the tendon fusion becomes evident (A). After the PT and triceps coxae (TCT) are dissected and folded away, the subtendinous bursa of the obturator internus *(green arrowhead pointing to the right)* and the piriformis bursa *(orange arrowhead pointing to the left)* become visible. The yellow arrowhead pointing upwards indicates PT–GMed fusion. GI, gemellus inferior; GMax, gluteus maximus; GS, gemellus superior; GT, greater trochanter; JC, Joint capsule of the hip; OI, obturator internus; SN, sciatic nerve.

Fifteen lower limbs with bisected pelvises from formalin‐embalmed cadavers (5 male, 10 female; 7 left and 8 right sides) were dissected. A fat pad overlying the PT and TCT was observed in 93% (14/15) of the cases (Figure 2A,B). Consistent (15/15) fusion of the PT with the TCT was observed, and in 93% (14/15) of the cases, the PT also fused with the gluteus medius tendon (Figure 3A). A bursa of piriformis was identified in 80% (12/15) of the cases, and a subtendinous bursa of obturator internus was found in 73% (11/15) (Figure 3B). Ten specimens had both subtendinous bursa of the obturator internus and the bursa of piriformis, and two specimens lacked either of these bursae. Table 2 summarizes the details of our findings.

### Ultrasound‐guided Injection

**FIGURE 4:**
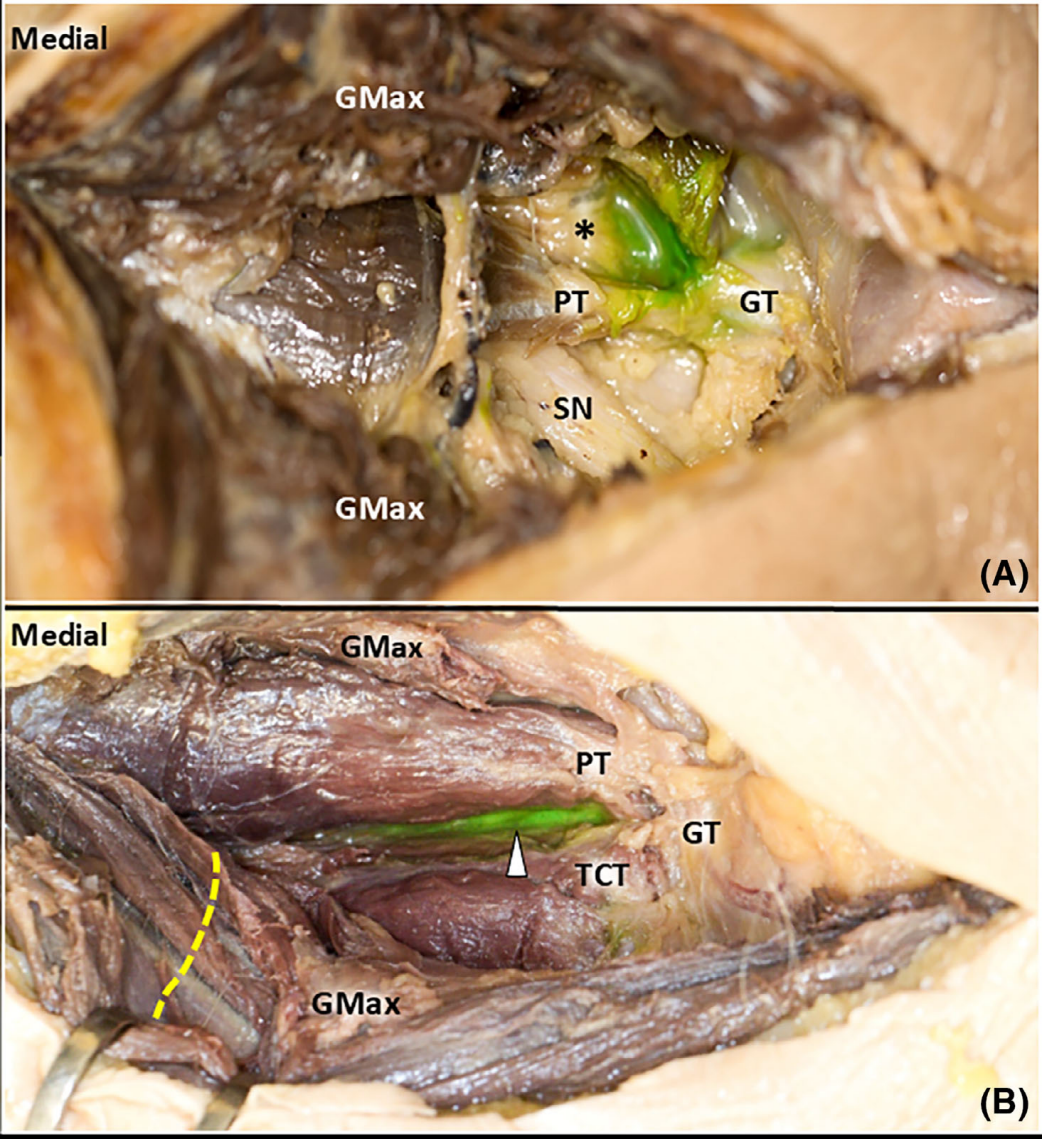
Cadaveric (Fix‐4‐Life method) features and postinjection dye localization. Dissection of a cadaveric specimen following the superficial/peritendinous injection (A). After the skin was removed, an incision was made along the fiber direction of the gluteus maximus (GMax), followed by retracting sections of the muscle using hooks. The anticipated location of the SN, beneath the GMax, is indicated by a dashed line. In both cases, the green latex dye was found inside the fat pad (*asterisk*) overlying the piriformis tendon (PT) and the TCT. After the deep/intrabursal injection, the dye (*arrowhead*) was found deep to the PT and triceps coxae in 86% of the cases (6/7) (B). GMed, gluteus medius; GS, gemellus superior; GT, greater trochanter; OI, obturator internus; SN, sciatic nerve; TCT, triceps coxae tendon.

Initially, one full body F4L cadaver (two sides) was injected with the intention of achieving a peritendinous injection. In both cases, the latex dye was found inside the fat pad covering the PT and TCT (Figure 4A), considered to be unsuccessful. The intrabursal technique was subsequently performed on four cadavers. One side of one cadaver was deemed unsuitable for injection due to raised skin caused by mechanical deformation during the embalming process. Of the seven injected sides, latex dye was identified deep to the PT and TCT in six cases (Figure 4B) and within the hip joint in one case. In specimen 1 (on the right side), the dye was also observed staining the sciatic nerve. The success rate for the deep/intrabursal technique was 86%. Detailed results are presented in Table 2.

## DISCUSSION

This study provides novel insights into the anatomy of the deep retrotrochanteric region and validates a pertinent targeted US‐guided injection technique. Our anatomical study revealed an unexpectedly consistent fusion of the PT with the triceps coxae and frequent fusion with the gluteus medius tendon, contrasting with Windisch et al.'s findings. In their study, the authors dissected 112 lower extremities and reported fusion of the PT with at least one part of the triceps coxae in only 42% of cases.^13^ They systematically described four possible PT insertion types, highlighting the complexity of these anatomical structures.

Our study identified the bursa of piriformis in 80% of the cases and the subtendinous bursa of obturator internus in 73% of cases. Bursitis could be significantly symptomatic in these regions, and validation of the deep/intrabursal US‐guided injection technique offers a promising approach in this sense. Our poor results regarding the superficial/peritendinous injections, with the latex dye consistently being found in the fat pad overlying the PT–TCT, emphasize the importance of precise needle placement. Of note, this fat pad was previously reported as a potential source of diagnostic pitfall, as its hypoechoic appearance can be misinterpreted as an effusion.^14^ The aforementioned findings suggest that superficial injections may be inadequate for addressing the underlying pathology in cases of PT–TCT tendinopathy when utilizing the methodologies employed in our study, specifically the use of a linear probe and embalmed cadavers.

We originally intended to study the superficial approach using more F4L cadavers; however, after the failure of the first two injections, we decided not to continue with injecting other cadaveric specimens and focused on the deep/intrabursal technique. Notably, this technique demonstrated an 86% success rate, which appears to be more effective in delivering the injectates to the target area. In specimen 3 (on the right side), in addition to the correct dye placement between the PT–TCT and the hip joint capsule, the dye was also found in the trochanteric bursa. We assume that this communication may be attributed to an anatomic connection, a tendon tear, or chronic inflammation in the hip region. In specimen 1 (on the right side), the dye was also observed staining the sciatic nerve, likely as the result of medial spread from the bursa. Given the potential risk of motor block in the sciatic nerve, the procedure may require post‐procedural precautions. In specimen 3 (on the left side), the latex dye was found within the hip joint, possibly due to an incompetent joint capsule or a technical issue during the injection, such as inserting the needle too deeply. Needless to say, the retrotrochanteric region contained an unusually high volume of free fixation solution that made the procedure more difficult. We speculate that on living patients, the accuracy rate could be even higher. Importantly, because the gluteal region is a typical site for subcutaneous fat storage, the adipose tissue of obese people would negatively influence accuracy.

Our results should be considered in the context of the study by Smith et al.,^4^ which shares certain methodological similarities to our study. In their study, the authors successfully demonstrated the feasibility of four injection techniques targeting three distinct sites of the obturator internus using US guidance. These sites included (1) the tendon sheath, (2) intramuscular injections within the muscle belly of the obturator internus, and (3) the obturator internus bursa. Notably, their focus was on more medial structures compared to our study. The tendon sheath technique specifically targeted the space between the sciatic nerve and the emergence of the obturator internus from the pelvis. Intramuscular injections were administered near the musculotendinous junction, and the obturator internus bursa was accessed between the obturator internus and the posterior ischium. It is important to note that the obturator internus bursa targeted by Smith et al.,^4^ referred to as the sciatic bursa in Synnnevedt's monograph,^8^ is distinct from subtendinous bursa of the obturator internus targeted in our study.

### Clinical Implications

There are anecdotal reports describing inflammation in the TCT and its associated bursae. More specifically, Hwang et al. reported two cases highlighting the magnetic resonance imaging features of obturator internus bursitis.^15^ In these cases, the authors identified fluid collection between the obturator internus tendon and the posterior surface of the ischium, in a region they referred to as the “obturator internus bursa.” However, this localization was more medial than the region of interest in our study. Notably, Sadigale et al. described an inflamed “obturator bursa” overlying the obturator internus tendon at its insertion site. In our study, we did not observe a bursa located superficially to the obturator internus tendon at its insertion.^16^ Regarding the bursa associated with the PT, the literature on its clinical significance is even more limited.

These findings highlight the terminological inconsistency in the literature regarding bursae in the retrotrochanteric region. To address this issue, we advocate for adopting the English transcription of Synnesvedt's original terminology. Further evidence supporting the existence of obturator internus tendinitis is suggested by symptomatic relief observed following targeted injections.^17^, ^18^

By targeting the PT–TCT and its associated deep bursae, this approach may provide relief for those experiencing pain in the retrotrochanteric region. Moreover, the application of this technique can also serve as a diagnostic tool to confirm the involvement of the PT–TCT and the surrounding structures in relevant patients. Tailored rehabilitation programs targeting piriformis/triceps coxae, identified as pain generators based on the injection results, can be effectively planned and implemented to potentially reduce the risk of recurrent injuries. A positive injection response could help build confidence for surgical release in refractory cases as this type of surgery would generally be very unpredictable in relieving pain.^19^

It is important to differentiate the retrotrochanteric pain syndrome from greater trochanteric pain syndrome (GTPS) due to their different anatomical locations and clinical presentations. GTPS primarily affects the large hip abductors, particularly the gluteus medius and minimus tendons. However, retrotrochanteric pain syndrome affects posterior hip external rotators, including the piriformis and the triceps coxae. The location of pain also differs between these conditions. GTPS typically presents with lateral pain around the greater trochanter, often exacerbated by activities such as lying on the affected side, climbing stairs, or walking. Retrotrochanteric pain syndrome, on the other hand, is characterized by pain that is more posterior to the greater trochanter, and often radiates deep into the buttock or posterior thigh. Symptoms are usually aggravated by prolonged sitting or movements that stretch the posterior hip structures. Understanding these distinctions is essential for prompt diagnosis and appropriate management.

### Limitations

Several limitations need to be acknowledged. Regarding the post‐injection dissection methodology, the presence of dye beneath the PT or TCT, identified by displacing the tendons with tweezers, was considered indicative of intrabursal placement. However, further dissection to precisely localize the dye within individual bursae or evaluate potential communication between them was not performed. Second, cadaveric specimens may not authentically simulate physiological conditions in real/living patients. The embalming process can alter tissue properties, potentially affecting the accuracy of the injection. Fresh specimens may have provided a more authentic and realistic experience for research compared to embalmed or fresh‐frozen ones. Importantly, overall low body mass index of the specimens may have made injections in the study technically easier than in a population with a higher prevalence of obesity. Another limitation is the absence of factors such as patient real‐time feedback during injection. Third, the sample size for the anatomical part of the study was limited to 15 bisected pelves/lower extremities. The study population was also predominantly female. Due to hormonal differences, females generally have greater fat deposition in the gluteal region compared to males. This thicker fat layer tends to absorb more US energy, which can result in reduced clarity of deeper structures and make precise needle targeting more challenging. Conversely, the gluteus maximus muscle, which must be traversed during injection, is typically less hypertrophic in females than in males. These anatomical differences may limit the applicability of our findings to male patients. When a significant fat or muscle layer is present, using a low‐frequency transducer can improve penetration, though this comes at the cost of image resolution.

### Conclusion

This study enhances the understanding of the relationships between the tendinous structures of the deep retrotrochanteric region and establishes a validated US‐guided PT–TCT injection. Its high cadaveric success rate (86%) suggests that this approach may offer significant therapeutic benefits, particularly for patients with recalcitrant chronic gluteal pain. Future research should report on pathologic findings of the PT and TCT/bursae and correlate with patients experiencing retrotrochanteric pain syndrome. In addition, future studies should report on the efficacy of this injection technique in patients with retrotrochanteric pain syndrome, as well as compare it with previously described injection techniques.

## FUNDING INFORMATION

Supported by Ministry of Health, Czech Republic – conceptual development of research organization 00064165, General University Hospital in Prague.

## PATIENT CONSENT

Informed consent had been obtained from donors for scientific and educational purposes prior to death, and approval was obtained from the Institute of Anatomy, First Faculty of Medicine, Charles University, Prague.

## DISCLOSURE

None.
